# HDL/ApoA-1 infusion and ApoA-1 gene therapy in atherosclerosis

**DOI:** 10.3389/fphar.2015.00187

**Published:** 2015-09-01

**Authors:** Kuang-Yuh Chyu, Prediman K. Shah

**Affiliations:** Division of Cardiology, Oppenheimer Atherosclerosis Research Center, Cedars-Sinai Heart Institute, Cedars-Sinai Medical CenterLos Angeles, CA, USA

**Keywords:** atherosclerosis, HDL, ApoA-1, gene therapy

## Abstract

The HDL hypothesis stating that simply raising HDL cholesterol (HDL-C) may produce cardiovascular benefits has been questioned recently based on several randomized clinical trials using CETP inhibitors or niacin to raise HDL-C levels. However, extensive pre-clinical data support the vascular protective effects of administration of exogenous ApoA-1 containing preβ-HDL like particles. Several small proof-of-concept clinical trials using such HDL/ApoA-1 infusion therapy have shown encouraging results but definitive proof of efficacy must await large scale clinical trials. In addition to HDL infusion therapy an alternative way to exploit beneficial cardiovascular effects of HDL/ApoA-1 is to use gene transfer. Preclinical studies have shown evidence of benefit using this approach; however clinical validation is yet lacking. This review summarizes our current knowledge of the aforementioned strategies.

## Epidemiology and HDL

Epidemiologic studies have, in general demonstrated an inverse relationship between the level of HDL-C and the risk of atherosclerotic cardiovascular diseases (ACVD) (Miller and Miller, 1975; Gordon et al., 1977; Miller et al., 1977). If such a relationship were causal then it has been estimated that for every 1 mg/dl increase in HDL-C level, there would have been a decrease of 2–3% in coronary heart disease risk in men and women, respectively (Gordon et al., 1989). Given the positive correlation between the level of LDL cholesterol (LDL-C) and ACVD risk and the success of lowering LDL-C level pharmacologically or by other means to reduce ACVD events, the medical community had embraced the idea that raising HDL-C level would similarly lead to clinical benefit supporting the so called HDL hypothesis. This concept has been supported by the demonstration that HDL and its ApoA-1 exhibit several biological actions that could favorably affect athero-thrombosis. Furthermore, transgenic over-expression of ApoA-1 gene, direct infusion of plasma derived or recombinant wild type or mutant ApoA-1 and gene transfer of other HDL associated proteins have been shown to be athero-protective in various experimental and limited clinical settings, thereby justifying current focus on HDL based therapies (Shah et al., 2001a,b).

Based on this notion, several clinical trials have been conducted to test the clinical efficacy of HDL-C raising therapy. Cholesteryl ester transfer protein (CETP) inhibitors such as torcetrapib and dalcetrapib are capable of raising HDL-C level by 72 and 30%, respectively. In the ILLUMINATE trial, Torcetrapib plus Atorvastatin group experienced a significant excess in cardiovascular and non-cardiovascular mortality despite a marked increase in HDL-C level and a significant additional lowering of LDL-C level compared to recipients of Atorvastatin alone (Barter et al., 2007). Similarly, torcetrapib plus atorvastatin did not affect the progression of atherosclerosis of carotid or coronary arteries compared to Atorvastatin alone (Bots et al., 2007; Nicholls et al., 2008). In the dal-OUTCOMES trial, dalcetrapib, another CETP inhibitor without known off target adverse effects failed to reduce the risk of recurrent cardiovascular events in patients who had a recent acute coronary syndrome (ACS) (Schwartz et al., 2012). Currently there are two additional CETP inhibitors, Evacetrapib (clinical trial identifier: NCT01687998) and Anacetrapib (clinical trial identifier: NCT00685776), being tested in phase III randomized clinical trials. These new CETP inhibitors produce very large (>100%) increases in HDL-C as well as large reductions in LDL-C (>20%) and the results of these trials will provide further evidence in favor or against CETP inhibition. However, the mechanism of any benefit, if found, may be difficult to assign to HDL-C increase when there is a concomitant marked reduction in LDL-C. Daily dose of niacin 1–3 gm raises HDL-C level with concomitant lowering of plasma triglycerides, LDL-C and possibly lipoprotein(a). The efficacy of niacin in reducing ACVD events on top of effective statin therapy and LDL-C lowering has been investigated in two clinical trials. In the AIM-HIGH trial, treatment with extended-release niacin did not offer incremental clinical benefit when given to patients on statin therapy with baseline LDL-C level < 70 mg/dl (Boden et al., 2011). This trial was prematurely terminated because of futility. In the HPS2-THRIVE trial, adding extended-release niacin in combination with a flush inhibitor, laropiprant, to statin based therapy did not confer clinical benefit in over and above statin alone but instead increased the rate of adverse events (HPS2-THRIVE Collaborative Group, 2013; Landray et al., 2014). Fibrates are another class of agent that raise HDL-C and also lower triglycerides. Fenofibrate was tested in the ACCORD study in patients with type 2 diabetes on statin therapy and failed to show reduction of AVCD events when compared to statin therapy alone (Ginsberg et al., 2010). A full discussion of the pros and cons of these clinical trials is beyond the focus of this review and the reader is referred to several excellent reviews that have addressed these issues (Kingwell et al., 2014; Tariq et al., 2014; Tuteja and Rader, 2014).

These negative trials have challenged the HDL-C hypothesis and many tangible reasons have been proposed to explain such failures. These trials were conducted in patients on background of effective statin treatment with well controlled LDL-C levels, which could confound the efficacy of HDL-C raising therapy and potentially the interpretation of the trial results. Torcetrapib was shown to raise blood pressure through activation of the renin-angiotensin-aldosterone pathway and such off target effect may have contributed to its failure. It also remains unclear whether the failure of niacin in HPS-2 study was related to the addition of laropiprant, a PG DP1 receptor antagonist, to the niacin. It has also been argued that failure of AIM-High trial may have reflected a flaw in the trial design, i.e., underpowered to show a small benefit. Experts in the field have also questioned if simply raising the HDL-C level pharmacologically could translate to athero-protective effects and have introduced the concept of quality or the functionality of HDL and its protein components, especially ApoA-1, in mediating the protective effect of HDL (Gadi et al., 2013; Kypreos et al., 2013; Barylski et al., 2014; Karavia et al., 2014). Others have re-examined the relationship between HDL-C level and ACVD risk using Mendelian randomization analysis. This analysis utilized screening the entire genome for thousands of single nucleotide polymorphisms (SNPs) in large cohorts to identify statistical relationships between clinical outcomes and alleles associated with putative variables to discover etiological factors in diseases (Kingwell et al., 2014). Voight et al. reported that carriers of a SNP in endothelial lipase gene (LIPG Asn396Ser) had higher HDL-C level but similar levels of other lipid and non-lipid risk factors for myocardial infarction compared with non-carriers. This difference in HDL-C level was predicted to decrease risk of myocardial infarction by 13% but in fact was not associated with such risk reduction. Additionally a 1 SD increase in HDL-C level due to genetic score was not associated with risk of myocardial infarction, opposite to the observation from population epidemiology that an increase of 1 SD in HDL-C level was associated with reduced risk of myocardial infarction (Voight et al., 2012). Another study using the same methodology also failed to find a causal effect of HDL-C level on coronary heart disease risk (Holmes et al., 2015). These genetic epidemiology studies further challenge the concept of using HDL-C level as treatment target to test HDL-C hypothesis.

Given the lack of support of HDL-C hypothesis by raising HDL-C level pharmacologically, the quality and function of HDL and its ApoA-1 becomes the current focus of intensive research and potential future therapeutic targets. HDL is a complex molecule that is heterogeneous in size and compositions which may determine the functionality of the HDL particles (Tsompanidi et al., 2010; Kypreos et al., 2013). Numerous pre-clinical and clinical studies have reported diverse HDL functions including reverse cholesterol transport, antioxidant, anti-inflammatory properties, and immune-modulatory effect (see below).

## Mechanistic basis for beneficial effects of HDL and ApoA-1

### Stimulation of reverse cholesterol transport

One of the principle actions of ApoA-1 is to stimulate reverse cholesterol transport to mobilize free cholesterol from the peripheral tissues such as lipid laden macrophages and carry the mobilized lipid to liver for uptake and elimination in feces through biliary sterols. Free cholesterol and phospholipid from the macrophage is transferred to lipid poor ApoA-1 discoid particles by ABCA1 transporter and to larger spherical HDL particles by ABCG1 transporter in the initial steps of the reverse cholesterol transport; the free cholesterol is then esterified by lecithin cholesterol acyl transferase leading to formation of spherical HDL_2_ and HDL_3_ particles. HDL then delivers cholesterol ester to the liver via SRB1 receptors or exchanges cholesterol ester for triglycerides from VLDL and LDL particles, an exchange facilitated by CETP. The cholesterol ester transferred to LDL and VLDL is then targeted for hepatic uptake via the LDL receptor pathway or accumulates in the arterial wall via subendothelial retention. Thus, mobilization of free cholesterol from arterial walls by ApoA-1 and HDL contributes to an anti-atherogenic effect (Shah et al., 2001a).

### Anti-inflammatory and immuno-modulatory effects

Inflammation and immune activation play an important role in atherogenesis. Several reports have delineated the anti-inflammatory properties of ApoA-1 *in vitro*. ApoA-1 inhibited IL-1β and TNF-α production from T-cell activated monocytes (Hyka et al., 2001). ApoA-1 also diminishes neutrophil adhesion, oxidative burst and degranulation from activated neutrophils; further implicating the ability of ApoA-1 to modulate inflammation (Liao et al., 2005). Additionally ApoA-1 affects dendritic cell maturation by rendering them less responsive to anti-CD40 antibody and interferon-γ stimulation, possibly via increased IL-10 and PGE_2_ production (Kim et al., 2005). HDL may also promote migration of dendritic cells out of vascular lesions into regional lymph nodes (Angeli et al., 2004), possibly by neutralizing the dendritic cell immobilizing effects of phospholipids generated during LDL oxidation. HDL also attenuates the inhibitory effects of inflammation on reverse cholesterol transport. Recent experimental observations have shown that ABCA1 and ABCG1, critical players in the initial steps of reverse cholesterol transport from macrophages and critical regulators of HDL particle formation, play an important role in suppressing atherosclerosis associated leukocytosis by inhibiting bone-marrow derived progenitor cells (Yvan-Charvet et al., 2010).

### Anti-oxidant and endothelial protective effects

HDL inhibits LDL oxidation, scavenges toxic phospholipids from oxidized LDL and protects vascular smooth muscle cells and endothelial cells from damaging effects of oxidized LDL. The anti-oxidant effects of HDL have been attributed to Paraoxonase (PON), Platelet Activating Factor Acetylhydrolase (PAF-AH) carried by HDL (Shah et al., 2001a,b).

HDL protects endothelial cell functions by prevention of oxidation of LDL and its adverse endothelial effects. It also attenuates endothelial dysfunction from dyslipidemia and atherosclerosis by SRB-1 dependent induction of eNOS, attenuates endothelial cell apoptosis, and stimulates endothelial reparative capacity (Kimura et al., 2006; Seetharam et al., 2006; Mineo and Shaul, 2007; Pu and Liu, 2008). HDL from healthy subjects can stimulate endothelial nitric oxide production during endothelial repair processes (Besler et al., 2011) whereas HDL from patients with atherosclerotic vascular diseases or type II diabetes mellitus loses such function (Besler et al., 2011).

### Antithrombotic effects

HDL inhibits platelet aggregation, activates protein c and inhibits assembly of the prothrombinase complex on anionic phospholipid surfaces (Oslakovic et al., 2009). Native HDL from human also significantly impaired OxLDL-induced platelet aggregation and adhesion, decreased both the formation of reactive oxygen species and CD40L expression (Badrnya et al., 2013). Intravenous infusion with a reconstituted HDL (CSL-111) in individuals with type II diabetes mellitus resulted in a >50% reduction in the *ex vivo* platelet aggregation response, possibly via reduction of the cholesterol content of platelet membranes (Calkin et al., 2009). A recent report that elevated HDL-C levels are associated with improved fibrin clot lysis also supports the antithrombotic effects of HDL (Zabczyk et al., 2013).

### Pancreatic beta cell protective effects and improving glucose intolerance

Insulin resistance and type II diabetes are often associated with reduced levels of HDL-C. HDL infusion was shown to improve pancreatic beta cell function in type 2 diabetic patients. HDL may also protect against diabetes by reducing stress induced pancreatic beta cell apoptosis. Furthermore, ABCA1 and ABACG1 mediated anti-inflammatory effects of HDL may attenuate islet cell inflammation that is implicated in development of type 2 diabetes (Fryirs et al., 2009, 2010; Kruit et al., 2010). ApoA-1, in lipid free form or complexed with phospholipid, improved insulin sensitivity with decreased systemic and hepatic inflammation in mice fed high-fat diet (McGrath et al., 2014). ApoA-1_Milano_ has similar effect as well (Stenkula et al., 2014).

## Pre-clinical and clinical studies of HDL/ApoA-1/ApoA-1_*Milano*_ infusion

### Intravenous administration of homologous HDL

The effects of *in vivo* administration of HDL on the development of aortic fatty streaks were first tested in cholesterol-fed rabbits. This tested the concept of using exogenous HDL as a physiological acceptor for cholesterol from peripheral tissues. Rabbits receiving weekly infusion of homologous HDL-VHDL protein isolated from normal rabbit plasma developed smaller fatty streak lesions in the aortic intimal surface with reduced deposition of total and free cholesterol, esterified cholesterol, and phospholipids in the vessel wall when compared to control group receiving saline administration (Badimon et al., 1989). The same investigators later reported that administering exogenous HDL regressed the established fatty streak lesions in cholesterol fed rabbits with reduced total cholesterol, esterified cholesterol and phospholipids in aortic wall, suggesting increased reverse cholesterol transport (Badimon et al., 1990). Interestingly infusion of exogenous HDL did not alter circulating HDL-C level (Badimon et al., 1990). Taken together, these findings provided the initial support for the concept that the functionality of HDL plays a more important role to reduce atherosclerosis than the circulating level of HDL-C. Most plasma HDL-C exists in spherical HDL_2_ and HDL_3_ with a smaller percentage of HDL-C transported by small and discoidal particles with pre-β electrophoretic mobility (preβ-HDL) (Conca and Franceschini, 2008). Lipid poor apolipoproteins, mainly ApoA-1, are the major cholesterol acceptors for ABCA1 in the early steps of reverse cholesterol transport pathway. Hence investigators have been developing strategies to infuse purified ApoA-1 or synthetic HDL made of a purified ApoA-1 (wild type or mutant) complexed with a phospholipid.

### Intravenous infusion of ApoA-1_*Milano*_

ApoA-1_Milano_ is a naturally occurring mutant of ApoA-1 (Arg173Cysteine) carried by a small number of inhabitants of Limone sul Garda. The carriers are heterozygous for the mutation and have an unexpectedly low prevalence of cardiovascular disease and a family history of longevity despite very low HDL-C and ApoA-1 levels (Sirtori et al., 2001).

Since the early 1990's we have been investigating the vascular protective effects of recombinant ApoA-1_Milano_ complexed with a phospholipid carrier in experimental models. We first reported the striking athero-protective and anti-inflammatory effects of intravenous infusion of r-ApoA-1_Milano_ in cholesterol fed rabbits undergoing balloon injury of their ileofemoral arteries to accelerate lesion formation (Ameli et al., 1994). These results were later replicated by Soma et al in a perivascular carotid injury model in rabbits (Soma et al., 1995). Subsequently our laboratory reported that frequent intravenous injections of r-ApoA-1_Milano_ would also halt progression of atherosclerosis in ApoE^−∕−^ mice while reducing lipid and macrophage content in lesions (Shah et al., 1998). In yet another study involving ApoE^−I^ mice, we demonstrated that a large single IV dose of r-ApoA-1_Milano_ would rapidly deplete lipid and macrophages from atherosclerotic lesions within 48 h indicating the ability of this intervention to rapidly mobilize tissue cholesterol (Shah et al., 2001c). Similar findings were later reported by Chiesa et al in a rabbit model of carotid plaque (Chiesa et al., 2002). Additional work in our laboratory also demonstrated that r-ApoA-1_Milano_-phospholipid complex could reverse endothelial dysfunction in hypercholesterolemic apoE^(−*po*)^ mice (Kaul et al., 2004) and inhibit in-stent stenosis in porcine coronary arteries (Kaul et al., 2003).

Such beneficial plaque stabilizing effect with short-term treatment of ApoA-1_Milano_-phospholipids complex was also demonstrated later by other investigators in a rabbit atherosclerosis model with two infusions 4 days apart (Ibanez et al., 2008) or five treatments every 4 days (Parolini et al., 2008). The acute effect of short-term therapy with recombinant ApoA-1_Milano_ suggests the potential clinical application for acute plaque stabilization in patients with ACS.

Based on the body of work described, rApoA-1_Milano_ PC complex was developed as a therapeutic intervention for clinical testing by Esperion therapeutics (ETC 216). ETC-216 (Esperion Therapeutics, Ann Arbor, Mich) was tested in a small proof of concept clinical trial in which sequential intravascular ultrasound studies done before and after 5 weekly infusions of ETC-216 or placebo. The study showed rapid coronary plaque regression with rApoA-1_Milano_ (Nissen et al., 2003). However, subsequent clinical development was delayed by several years due to manufacturing difficulties and contamination from host derived proteins. Now a clean manufacturing process has been developed to produce the rApoA-1_Milano_ without contamination by host derived proteins and this new material, called MDCO-216 (The Medicines Company) has completed a phase 1 human trial without problems and phase 2 trials are being planned for later part of 2015 (Tardif, 2010).

A Canadian company, SemBioSys Genetics Inc, used a bio-pharming technique to produce genetically modified safflower, *Carthamus tinctorius*, that concentrates ApoA-1_Milano_ in the oil of their seeds (Nykiforuk et al., 2011). This plant engineered ApoA-1_Milano_ lacked first two amino acids (Des1,2 ApoA-1_Milano_) compared to E. Coli derived rApoA-1_Milano_ and appeared to be functionally similar to E. Coli derived recombinant ApoA-1_Milano_. Pre-clinical experiments in our laboratory using Des1,2 ApoA-1_Milano_ have shown promising results (data presented at Annual Scientific Sessions of the American College of Cardiology in 2010 as an abstract; http://content.onlinejacc.org/article.aspx?articleid=1141161). Unfortunately Des1,2 ApoA-1_Milano_ did not enter any clinical testing because SemBioSys Genetics ceased operation in 2012 due to financial reasons and bankruptcy.

### Intravenous infusion of wild type ApoA-1

Miyazaki et al. purified rabbit ApoA-1 from pooled plasma of 500 rabbits and demonstrated intravenous infusion of such purified homologous ApoA-1 was able to slow the progression of atherosclerosis in cholesterol-fed rabbits (Miyazaki et al., 1995).

The idea of infusing plasma derived human ApoA-1 into humans was tested in the 1990s. Lipid free ApoA-1 infusion into 6 men with low HDL-C level was able to increase plasma total ApoA-1 concentration without eliciting adverse effects and such increase was confined to the pre-beta region in the plasma (Nanjee et al., 1996). The same group of investigators subsequently showed infusion of ApoA-1-phosphatidylcholine complex into healthy men increased the intravascular production of small pre-β HDLs *in vivo* with associated increase in efflux and esterification of unesterified cholesterol (Nanjee et al., 1999). The small pre-β-HDL generated in plasma using this approach was able to cross endothelium into tissue fluid and promote efflux of unesterified cholesterol from peripheral cells (Nanjee et al., 2001). Infusion of ApoA-1-phosphatidylcholine complex into hypercholesterolemic patients rapidly normalized endothelium dependent vasodilation as demonstrated by forearm venous occlusion phethysmography (Spieker et al., 2002) but such strategy failed to demonstrate similar vasodilatory benefit in patients with ACS (Chenevard et al., 2012).

The efficacy of ApoA-1 infusion in patients with ACS was further tested by infusing purified wild type ApoA-1 from human plasma linked to soybean phosphatidylcholine (CSL-111, CSL Behring) at 40 mg/kg/infusion in the ERASE trial. Five weekly infusions did not show a significant difference in coronary atheroma volume in comparison to placebo (primary end point); however when compared to pretreatment baseline, CSL-111 recipients showed more regression in contrast to the placebo arm (Tardif et al., 2007). The 80 mg/kg/infusion regimen was abandoned because of hepatic toxicity even though in a separate study a single infusion of 80 mg/kg favorably reduced the expression of inflammatory marker and lipid content in the femoral artery plaque and raised HDL-C level and cholesterol efflux capacity of apoB-depleted plasma (Shaw et al., 2008).

CSL-112 is a successor of CSL-111 with an enhanced ability to accept cholesterol from ABCA1 (Diditchenko et al., 2013). When infused into human subjects, CSL-112 infusion was safe, well tolerated and produced increases in ApoA-1 concentration in a dose-dependent manner and remained elevated for 3 days without evidence of major organ toxicity or immunogenicity (Easton et al., 2014). In another study, infusion of CLS-112 into healthy human subjects increased HDL-C level and preferentially ABCA-1 dependent cholesterol efflux capacity (Gille et al., 2014). Based on these findings, CSL-112 will be tested in a phase 2b randomized, placebo-controlled, dose-ranging trial (ClinicalTrials.gov Identifier: NCT02108262) to investigate the safety and tolerability of multiple dose administration of CSL-112 in patients with ACS with major adverse cardiovascular events as its secondary end-point.

CER-001 is another engineered pre-β HDL mimic consisting of recombinant human ApoA-1 and 2 different phospholipid carriers. In pre-clinical study, CER-001 enhanced reverse lipid transport, reduced vascular inflammation, and promoted regression of atherosclerosis in hypercholesterolemic LDL receptor deficient mice (Tardy et al., 2014). Despite these beneficial effects in animal models, when tested in patients with ACS, CER-001 failed to demonstrate any plaque-reducing effect on coronary atherosclerosis as measured by IVUS and QCA when compared with placebo in the randomized CHI-SQUARE trial (Tardif et al., 2014). On the other hand, CER-001 was able to stimulate cholesterol mobilization and reduce artery wall dimension and inflammation in patients with the orphan disease familial hypoalphalipoproteinemia (Kootte et al., 2015). In another orphan disease, homozygous familiar hypercholesterolemia, bi-weekly infusion of CER-001 infusions significantly reduced carotid mean vessel wall area and mean vessel volume at 6 months as assessed by carotid MRI in the MODE (Modifying Orphan Disease Evaluation) study (http://www.eas-society.org/news-from-late-breaking-session-2.aspx, EAS 2014).

### Intravenous re-infusion of endogenous delipidated HDL

Based on the hypothesis that lipid poor ApoA-1 particles are particularly effective in stimulating ABCA1 mediated reverse cholesterol transport, the idea of re-infusing *ex vivo* delipidated endogenous plasma HDL was tested in a small human trial (LS-001, Lipid Sciences Selective Delipidation Trial). Eligible patients with ACS scheduled for cardiac catheterization with a nonobstructive atheroma in ≥1 native coronary arteries were randomized to HDL delipidation or control, and subjected to apheresis/reinfusion for seven sessions each, 1 week apart. IVUS evaluation of the target vessel showed a non-statistically significant coronary plaque regression as measured in patients receiving re-infusion of autologous plasma containing delipidated HDL (Waksman et al., 2010).

### Summary

Some, but not all, experimental studies have suggested that ApoA-1_Milano_ has more potent athero-protective effects than wild type ApoA-1 (Wang et al., 2006; Lebherz et al., 2007; Feng et al., 2009; Ibanez et al., 2012). Overall, the strategy of short term infusion of synthetic HDL (containing ApoA-1_Milano_ or wild type ApoA-1) is a promising pharmacological approach for rapid plaque remodeling and stabilization. Table 1 summarizes the results from currently available clinical studies using this strategy that could later be sustained with the use of orally effective LDL lowering and possibly HDL function improving agents or even possibly repeated infusions.

**Table 1:** **Clinical studies using HDL/ApoA-I infusion as therapeutic agent**.

**Formulation**	**Name of agent**	**Tested patient population**	**Primary outcome**
Recombinant ApoA-I_Milano_/phospholipid complex	ETC-216	Acute coronary syndrome	Coronary atherosclerotic plaque regression in the treatment group
Purified wild type ApoA-I from human plasma complexed with phosphatidylcholine	CSL-111	Acute coronary syndrome	Coronary atherosclerotic plaque regression in the treatment group
	CSL-111	Patients with claudication scheduled for percutaneous revascularization	Reduction of VCAM-1 expression and lipid content in the plaque; increase in HDL-C level and capacity of cholesterol efflux
Purified wild type ApoA-I from human plasma complexed with phosphatidylcholine	CSL-112	Healthy subjects	Dose-dependent increase in ApoA-I concentration without organ toxicity or immunogenicity
	CSL-112	Healthy subjects	Increase in ABCA-I dependent cholesterol efflux
	CSL-112	Acute coronary syndrome	Ongoing phase 2b trial to evaluate hepatic and renal safety and tolerability of CSL112
Recombinant human ApoA-I complexed with phospholipid	CER-001	Acute coronary syndrome	No change on coronary atherosclerotic plaque volume
	CER-001	Familial hypoalpha-lipoproteinemia	Stimulation of cholesterol mobilization and reduction of artery wall dimension and inflammation
	CER-001	Homozygous familiar hypercholesterolemia	Reduction of carotid mean vessel wall area and volume
Delipidated autologous plasma HDL	LS-001	Acute coronary syndrome	Non-significant regression of coronary atherosclerosis plaques in the treatment group

## ApoA-1 and ApoA-1_*Milano*_ gene therapy

Although intravenous infusion of synthetic ApoA-1 (wild type or Milano mutant) is potentially safe for clinical application, it is limited for clinical use due to the high cost of large scale production of ApoA-1 and need for repeated intravenous administration. Therefore, gene therapy could be an alternative approach to exploit athero-protective effects of ApoA-1 or ApoA-1_Milano_ for its possible long-term effect. We have previously shown that transplantation of bone marrow transduced with a retroviral vector containing a macrophage specific promoter and ApoA-1_Milano_ gene significantly reduces atherosclerosis and plaque inflammation in hyperlipidemic mice despite low levels of circulating levels of transgene (Wang et al., 2006). In this study we also demonstrated substantially superior athero-protective effects of ApoA-1_Milano_ gene transfer compared to wild type ApoA-1 gene transfer supporting the possible gain of function nature of the Milano mutation. We have recently shown that a single intravenous injection of AAV8 encoding ApoA-1_Milano_ gene produces significant inhibition of atherosclerosis progression in hyperlipidemic mice (Tian et al., 2015). Furthermore, using a model of established advanced atherosclerosis in mice, we have also shown that the combination of low fat diet and AAV8 mediated ApoA-1_Milano_ gene transfer produces robust regression of atherosclerosis in genetically engineered mice (In Press: Journal of Clinical Pharmacology and Therapeutics). Several other pre-clinical studies have also demonstrated that effective transfer of genes encoding ApoA-1 or ApoA-1 mutants using viral vectors produce significant reduction and or promote regression of atherosclerosis (Pászty et al., 1994; Tangirala et al., 1999; Ishiguro et al., 2001; Major et al., 2001; Su et al., 2003), however clinical translation of such studies has not been reported to date.

For clinical application of gene therapy for atherosclerosis to become a reality, such strategy needs to overcome the following challenges—these have largely to do with the development of high efficiency, safe, non-immunogenic, scalable vectors that can lead to stable and long term transgene expression in the host without provoking adverse immunologic or non-immunologic complications. Recent progress in recombinant AAV technology is encouraging in this regard (Asokan and Samulski, 2013; Dismuke et al., 2013; High et al., 2014; Kotterman and Schaffer, 2014).

## Perspectives

In this review, we have discussed many approaches to exploit the well-established vascular protective effects of HDL and its major protein ApoA-1 with promising results in both pre-clinical and limited clinical studies. Once proven effective and safe in humans, they will be valuable additions to our current pharmacological armamentarium to treat atherosclerotic cardiovascular disease and its associated complications. The HDL-C hypothesis needs to be reinvented into the HDL hypothesis with emphasis on composition and functionality of HDL particles rather than HDL-C levels.

### Conflict of interest statement

The authors declare that the research was conducted in the absence of any commercial or financial relationships that could be construed as a potential conflict of interest.
